# Perceived red tape and occupational commitment among primary care physicians: a mixed-methods study of the mediating role of public service motivation

**DOI:** 10.3389/fpubh.2026.1855480

**Published:** 2026-07-08

**Authors:** Huichen Chang

**Affiliations:** School of Public Administration, Shandong Normal University, Jinan, China

**Keywords:** mixed methods, occupational commitment, primary care physicians, public service motivation, red tape

## Abstract

**Introduction:**

Occupational commitment among primary care physicians (PCPs) is critical for sustaining primary healthcare workforce stability, yet its institutional determinants remain insufficiently understood. This study examined whether perceived red tape is associated with occupational commitment and whether public service motivation (PSM) mediates this relationship.

**Methods:**

A convergent parallel mixed-methods design was employed. Quantitative data were collected from 306 PCPs across 36 primary healthcare facilities in Shandong Province, China. Confirmatory factor analysis and structural equation modeling were used to test the mediation model, with bias-corrected bootstrap confidence intervals and supplementary robustness checks. Qualitative data were obtained from semi-structured interviews with 36 PCPs and analyzed using thematic analysis.

**Results:**

Perceived red tape was negatively associated with PSM (*b* = −0.131, *p* = 0.013; *β* = −0.177), while PSM was positively associated with occupational commitment (*b* = 0.313, *p* < 0.001; *β* = 0.541). The indirect effect through PSM was statistically significant (*b* = −0.041, *p* = 0.047, 95% CI [−0.095, −0.010]), whereas the residual direct effect was not statistically significant after PSM was included. Qualitative analysis identified three forms of red tape: misaligned clinical regulations, repetitive documentation requirements, and administrative arrangements detached from clinical practice. These burdens were described as constraining autonomy, competence, and relatedness, which may help explain the motivational pathway.

**Conclusion:**

Perceived red tape was associated with lower occupational commitment among PCPs, with PSM serving as an important motivational pathway. Policy reforms should reduce low-value administrative burdens while preserving necessary regulatory functions.

## 1 Introduction

Primary healthcare is central to equitable access to health services and to the effective functioning of health systems (1). Improving primary healthcare service capacity depends not only on infrastructure and financial investment, but also on governance arrangements and a stable, motivated workforce (2, 3). However, in China, primary care physicians (PCPs), who form the core of this workforce, face a high risk of turnover and withdrawal from the profession (4). Existing studies have shown that PCPs often work under weak financial incentives and limited opportunities for career development. Compared with specialists in higher-level hospitals, they often have less specialized roles and fewer opportunities to develop distinctive technical expertise (5). They often face greater barriers to cross-organizational mobility. Taken together, these conditions may be associated with lower attachment to the profession.

Occupational commitment refers to a positive identification with one’s profession and a willingness to continue fulfilling professional roles (6). It is a useful indicator of PCPs’ willingness to remain in the profession and stay engaged in their work (7).

Although existing studies have identified a range of antecedents of occupational commitment, such as job satisfaction and perceived organizational support (8), most have focused on individual- and organizational-level factors. Less is known about how institutional arrangements, especially administrative rules and compliance requirements, shape PCPs’ occupational commitment. This omission is important because public service work is embedded in institutional environments that define not only what professionals do, but also how they experience the meaning and value of their work. In public service organizations, institutional arrangements such as rules, procedures, and compliance requirements shape work attitudes (9). Red tape, as a typical source of institutional pressure (10), may be an important factor associated with occupational commitment.

Bozeman defined red tape as rules, procedures, and institutional arrangements that impose high compliance burdens but fail to achieve their intended functional goals (11). Subsequent studies have suggested that red tape is shaped not only by objective organizational conditions but also by individual perceptions (12). In other words, not all administrative tasks constitute red tape. Institutional theory helps explain why administrative requirements are evaluated not only as formal obligations, but also in terms of their alignment with professional roles. Rules and procedures shape role expectations and define what is regarded as appropriate and meaningful work (13). For PCPs, such role expectations are closely tied to clinical judgment, patient care, and public service. Accordingly, administrative tasks are more likely to be perceived as red tape when they require substantial compliance effort but are not seen as supporting clinical or public service purposes. Existing studies have shown that red tape has become a common challenge in health systems worldwide. Common forms of red tape include, but are not limited to, insurance prior authorization requirements and redundant medical documentation (14, 15). Previous research has shown that red tape has negative effects on individual and organizational outcomes, such as job performance, turnover intention, organizational performance, and client satisfaction (16–19). However, its relationship with occupational commitment has not been directly examined.

The association between red tape and occupational commitment may operate through individuals’ motivational systems. Primary healthcare services have strong public service attributes. The motivation of PCPs comes not only from external rewards but also from the pursuit of public values, such as serving patients and promoting social welfare (20). Public service motivation (PSM), defined as an individual’s intrinsic drive to promote public interest and social welfare (21), provides a useful perspective for understanding this relationship among PCPs. Previous studies suggest that PSM is not a stable personal trait but is influenced by the institutional environment (22). Self-determination theory distinguishes autonomous from controlled motivation. It argues that high-quality work motivation is supported when the basic psychological needs for autonomy, competence, and relatedness are satisfied, whereas need frustration may shift motivation toward controlled compliance (23, 24). From this perspective, PSM can be understood as an autonomous and identity-based form of motivation, because public-serving behavior is sustained when individuals personally endorse public values (25). Red tape may limit clinical autonomy, reduce opportunities to experience professional competence in patient care, and weaken the relational meaning of serving patients. By being associated with frustration of these basic needs, red tape may make it harder for PCPs to experience daily clinical work as a meaningful form of public service, thereby weakening PSM. When PSM is lower, PCPs may find it harder to experience clinical work as a self-endorsed way to realize public values. They may then evaluate the profession more through external conditions, such as rewards, workload, and career prospects (23). Because primary healthcare positions often provide relatively limited financial incentives and career development opportunities, this motivational shift may weaken emotional attachment to the profession and willingness to remain engaged, thereby reducing occupational commitment (5, 6).

Based on this theoretical reasoning, this study proposes a mediation model in which perceived red tape is negatively associated with occupational commitment, and PSM serves as the motivational pathway linking the two. Four hypotheses are proposed:

*H1*: Perceived red tape is negatively associated with occupational commitment among PCPs.

*H2*: Perceived red tape is negatively associated with PSM among PCPs.

*H3*: PSM is positively associated with occupational commitment among PCPs.

*H4*: PSM mediates the association between perceived red tape and occupational commitment.

**Figure 1:**
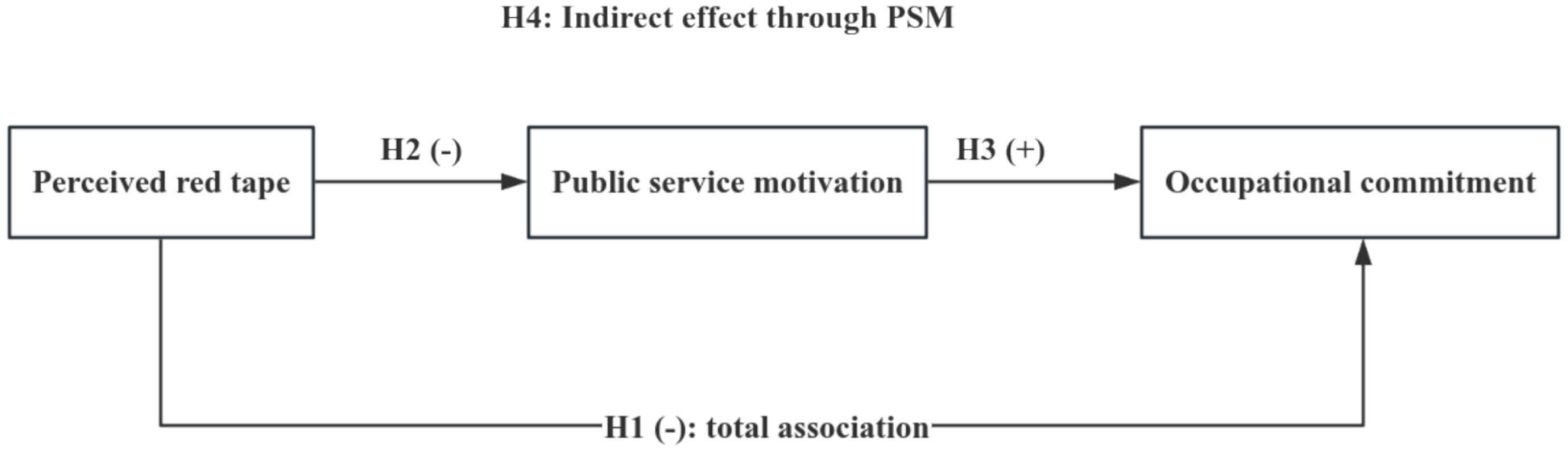
Conceptual model of perceived red tape, public service motivation, and occupational commitment.

This study makes three main contributions. First, it extends research on occupational commitment among PCPs by identifying perceived red tape as an institutional-level factor. Second, it links institutional theory and self-determination theory to explain how administrative burdens may be translated into motivational consequences. Third, by using a mixed-methods design, it combines quantitative evidence on the mediation pathway with qualitative evidence on how PCPs experience red tape in daily clinical work. The findings have implications for reducing low-value administrative burdens, improving institutional fit, and supporting PSM and occupational commitment in primary healthcare (see Figure 1).

## 2 Materials and methods

### 2.1 Study design

**Figure 2:**
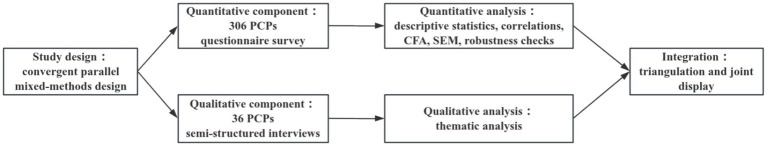
Flow diagram of the convergent parallel mixed-methods design.

A convergent parallel mixed-methods design was used. The quantitative component was used to examine the statistical mediation model proposed in this study, whereas the qualitative component was used to identify the main forms of perceived red tape among PCPs and to further elaborate how PSM functioned in the relationship between red tape and occupational commitment. In this way, the qualitative findings provided contextual detail and helped deepen understanding of how the observed relationships may unfold in practice. The overall study procedure is summarized in Figure 2.

### 2.2 Sample and procedures

#### 2.2.1 Quantitative sample and survey procedures

Quantitative data were drawn from a questionnaire survey conducted in primary healthcare facilities in Shandong Province, China. A multi-stage stratified cluster sampling method was used. Three prefecture-level cities, Yantai, Zibo, and Liaocheng, were selected to represent the eastern, central, and western parts of Shandong Province. Within each city, three districts or counties were randomly selected, and four primary healthcare facilities were then chosen from each district or county. A total of 36 facilities were included in the survey. The survey targeted PCPs on duty at the facilities on the day of data collection. In July 2023, trained investigators distributed the questionnaires, provided instructions, and obtained informed consent. After cases with missing values on key variables were excluded, 306 participants remained for analysis.

#### 2.2.2 Qualitative sample and interview procedures

Semi-structured interviews were conducted in the same 36 primary healthcare facilities included in the quantitative survey. Convenience sampling was used to recruit one PCP from each facility, resulting in a qualitative sample of 36 participants. Interviews were conducted in private settings to protect confidentiality and encourage candid discussion. An interview guide was used, and follow-up questions were asked when participants raised relevant issues. Each interview lasted approximately 30–60 min. With informed consent, the interviews were audio-recorded and supplemented with field notes. The recordings were subsequently transcribed verbatim for analysis.

### 2.3 Measures

#### 2.3.1 Quantitative measures

##### 2.3.1.1 Red tape

Red tape was measured using a three-item scale developed by Borry (26). The scale assesses respondents’ subjective perceptions of red tape in their organization. The items focus on inconvenience caused by rules that exceed actual needs, excessive time spent complying with organizational procedures, and formal requirements that hinder the achievement of meaningful work goals. All items were rated on a 5-point Likert scale (1 = strongly disagree, 5 = strongly agree). Higher scores indicate a higher level of perceived red tape. In this study, the scale showed good internal consistency, with Cronbach’s *α* = 0.893.

##### 2.3.1.2 Occupational commitment

Occupational commitment was measured using Blau’s eight-item scale (27), which assesses emotional attachment to the current profession and intention to remain in it. All items were rated on a 5-point Likert scale (1 = strongly disagree, 5 = strongly agree). Higher scores indicate a higher level of occupational commitment. In this study, the scale showed good internal consistency, with Cronbach’s *α* = 0.833.

##### 2.3.1.3 PSM

PSM was measured using an eight-item short scale revised by Bao on the basis of Kim’s cross-cultural PSM scale (28), which has been validated in the Chinese context and shown good reliability and validity in previous studies. The scale covers four dimensions: attraction to public policymaking, commitment to the public interest, compassion, and self-sacrifice. All items were rated on a 5-point Likert scale (1 = strongly disagree, 5 = strongly agree). Higher scores indicate a higher level of PSM. In this study, the scale showed good internal consistency, with Cronbach’s *α* = 0.935.

#### 2.3.2 Qualitative interview guide

A semi-structured interview guide was developed to correspond to the three core constructs in the quantitative model. The first question elicited PCPs’ concrete experiences of administrative burden and perceived red tape. The second question explored motivational orientations related to PSM, including participants’ views on serving patients, public welfare, and work motivation under limited external incentives. The third question elicited reflections on occupational identification and the perceived desirability of the medical profession. Because direct questions about emotional attachment to the profession may be susceptible to social desirability bias, participants were asked whether they would like their children to become doctors as an indirect prompt. The main interview questions included the following: (1) “Do you think primary healthcare staff have a heavy workload related to administrative tasks, such as meetings, form filling, and inspections or assessments? Have these tasks caused any difficulties or inconvenience for you?” (2) “Primary healthcare is different from hospitals. It emphasizes public welfare. Many services are low-cost or even free, and there are limited financial incentives. In this situation, what motivates you to continue your work?” (3) “Would you like your children to become doctors?”

### 2.4 Data analysis

#### 2.4.1 Quantitative analysis

Quantitative analyses were conducted using IBM SPSS Statistics 26.0 (Statistical Package for the Social Sciences) and Mplus version 8.3. Descriptive statistics were used to summarize the sample characteristics and the distributions of the main variables. Pearson correlation analysis was used to examine bivariate associations among perceived red tape, PSM, and occupational commitment. Harman’s single-factor test was conducted as a preliminary diagnostic test for common method bias.

The measurement model and hypothesized mediation model were then examined using Mplus 8.3. Confirmatory factor analysis (CFA) was first conducted to assess the measurement structure of perceived red tape, PSM, and occupational commitment. Perceived red tape was specified as a latent construct indicated by its three items. Given the multidimensional structure of PSM and the limited number of items within each dimension, four dimension parcels were created by averaging the two items corresponding to each PSM dimension, namely attraction to public policymaking, commitment to the public interest, compassion, and self-sacrifice. Occupational commitment was specified as a substantive latent construct indicated by eight items. Because the occupational commitment scale included three reverse-worded items, an orthogonal wording-method factor was specified for these items to account for potential wording effects. The CFA model was estimated using the maximum likelihood with robust standard errors (MLR) estimator.

After the measurement model had been evaluated, a latent-variable SEM was estimated to test the hypothesized mediation model. In this model, PSM was regressed on perceived red tape and covariates, and occupational commitment was regressed on perceived red tape, PSM, and the same covariates. The covariates included gender, age, work experience, professional title, staffing status, administrative position, education level, and marital status. The indirect, direct, and total effects were estimated using bias-corrected bootstrapped confidence intervals based on 5,000 bootstrap samples. Because bootstrap confidence intervals were requested, the SEM was estimated using the ML estimator. Given the cross-sectional design, the SEM results were interpreted as evidence of statistical mediation rather than causal mediation.

Several additional analyses were conducted to assess the stability of the quantitative findings. First, an observed-score mediation model was estimated using scale means. Second, a nonlinear specification was tested by adding the squared term of mean-centered perceived red tape. Third, interaction-based heterogeneity analyses were conducted by testing interactions between perceived red tape and administrative position, staffing status, gender, and work experience. Fourth, an alternative reverse-path model was estimated to examine whether the data also supported a reverse mediation pattern from occupational commitment to perceived red tape through PSM.

#### 2.4.2 Qualitative analysis

Qualitative data were analyzed using thematic analysis. The analysis combined inductive coding and theory-informed focused coding. First, the transcripts were read repeatedly to gain familiarity with the data. Open coding was then conducted to generate initial codes. Second, related codes were grouped into broader themes and subthemes. These themes captured the main forms of perceived red tape among PCPs and the ways in which red tape was experienced in relation to PSM and occupational commitment. Third, focused coding was conducted with reference to the red tape–PSM–occupational commitment pathway. Constant comparison across interviews and analytic memos were used to refine the themes and record coding decisions. Accounts that did not fully fit the main pathway were also examined when interpreting the qualitative findings. MAXQDA 2020 was used to support data management, coding, and thematic analysis.

#### 2.4.3 Mixed-methods integration

Quantitative and qualitative findings were integrated at the interpretation stage using a triangulation approach. The quantitative analysis tested the hypothesized statistical relationships among perceived red tape, PSM, and occupational commitment, whereas the qualitative analysis identified the concrete forms of perceived red tape and clarified how these institutional burdens were experienced by PCPs in daily practice. Integration focused on whether the qualitative themes converged with, elaborated, or qualified the quantitative mediation pattern. A joint display was developed to connect the main quantitative findings with corresponding qualitative themes and integration inferences. This integration was used to interpret the statistical associations in light of PCPs’ work experiences.

## 3 Results

### 3.1 Quantitative findings

#### 3.1.1 Common method bias test

Harman’s single-factor test was conducted to assess common method bias. The results showed that the first factor accounted for 39.06% of the total variance, which is below the commonly used threshold of 50%, suggesting that a dominant single-factor structure was not evident.

#### 3.1.2 Sample characteristics

Table 1 summarized the sociodemographic characteristics of the 306 PCPs. Overall, 56.9% were female, 67.7% were aged 30–49 years, and 71.2% had more than 10 years of work experience. Most participants were married (85.9%), held a bachelor’s degree (68.0%), and had junior or intermediate professional titles (70.0%). In addition, 86.6% had permanent public institution staffing, whereas only 17.0% reported holding administrative positions.

**Table 1:** Sociodemographic characteristics of PCPs (*N* = 306).

Variable	*n*	%
Gender		
Male	132	43.1
Female	174	56.9
Education		
College or below	95	31.0
Bachelor’s degree	208	68.0
Master’s degree or above	3	1.0
Age (years)		
≤29	47	15.4
30–39	92	30.1
40–49	115	37.6
≥50	52	17.0
Work experience (years)		
≤5	29	9.5
5–10	59	19.3
10–20	105	34.3
≥20	113	36.9
Marital status		
Married	263	85.9
Unmarried and others	43	14.1
Professional title		
None	35	11.4
Junior	100	32.7
Intermediate	114	37.3
Senior	57	18.6
Staffing status		
With staffing	265	86.6
Without staffing	41	13.4
Administrative position		
Yes	52	17.0
No	254	83.0

Values are descriptive statistics reported as *n* (%).

#### 3.1.3 Descriptive statistics and correlations

Table 2 presented the means, standard deviations, and Pearson correlations for red tape, PSM, and occupational commitment. The mean scores were 8.21 (SD = 3.367) for red tape, 34.11 (SD = 5.324) for PSM, and 30.37 (SD = 5.842) for occupational commitment. Red tape was negatively correlated with both PSM (*r* = −0.148, *p* < 0.01) and occupational commitment (*r* = −0.275, *p* < 0.01), whereas PSM was positively correlated with occupational commitment (*r* = 0.449, *p* < 0.01).

**Table 2:** Descriptive statistics of study variables (*N* = 306).

Variables	M ± SD	1	2	3
1 Red tape	8.21 ± 3.367	1.00		
2 PSM	34.11 ± 5.324	−0.148^**^	1.00	
3 Occupational commitment	30.37 ± 5.842	−0.275^**^	0.449^**^	1.00

Pearson correlation coefficients are reported. Two-tailed tests were used. **p < 0.01.

#### 3.1.4 Measurement model

CFA was conducted to evaluate the measurement model, which showed acceptable fit to the data, χ^2^ (84) = 199.816, *p* < 0.001, CFI = 0.938, TLI = 0.923, RMSEA = 0.067, 90% CI [0.055, 0.079], and SRMR = 0.074. The standardized factor loadings ranged from 0.788 to 0.936 for perceived red tape, 0.813 to 0.871 for the PSM dimension parcels, and 0.329 to 0.887 for the occupational commitment factor. The wording-method factor significantly loaded on the three reverse-worded occupational commitment items, indicating that accounting for wording effects improved model specification. Composite reliability (CR) and average variance extracted (AVE) indicated acceptable internal consistency and convergent validity, with CR values ranging from 0.844 to 0.908 and AVE values ranging from 0.427 to 0.742, exceeding commonly accepted thresholds (CR > 0.70, AVE > 0.40). Discriminant validity was further supported by heterotrait–monotrait ratio of correlations (HTMT) values ranging from 0.21 to 0.64, below the 0.85 threshold.

#### 3.1.5 Structural mediation model

The structural mediation model showed acceptable fit to the data, χ^2^(227) = 421.763, *p* < 0.001, CFI = 0.928, TLI = 0.914, RMSEA = 0.053, 90% CI [0.045, 0.061], and SRMR = 0.062. Perceived red tape was negatively associated with PSM (b = −0.131, SE = 0.052, *p* = 0.013; *β* = −0.177, *p* = 0.009), and PSM was positively associated with occupational commitment (*b* = 0.313, SE = 0.068, *p* < 0.001; *β* = 0.541, *p* < 0.001). After PSM and covariates were included, the residual direct path from perceived red tape to occupational commitment was not statistically significant (*b* = −0.048, SE = 0.031, *p* = 0.125; *β* = −0.113, *p* = 0.080). The model explained 11.2% of the variance in PSM and 35.2% of the variance in occupational commitment.

The bootstrapped indirect effect of perceived red tape on occupational commitment through PSM was statistically significant (*b* = −0.041, SE = 0.021, *p* = 0.047, 95% BC bootstrap CI [−0.095, −0.010]). The direct effect was not statistically significant (b = −0.048, SE = 0.031, *p* = 0.125, 95% BC bootstrap CI [−0.126, 0.001]), whereas the total effect was significant (b = −0.089, SE = 0.041, *p* = 0.031, 95% BC bootstrap CI [−0.192, −0.024]). These results indicate a statistically significant indirect pathway from perceived red tape to occupational commitment through PSM, while also showing that the residual direct association was not significant after PSM was included. Taken together, these findings provided qualified support for the hypothesized model. H1 was supported at the level of the total association rather than the residual direct association after PSM and covariates were included. Specifically, perceived red tape was negatively correlated with occupational commitment, and the total effect in the SEM was statistically significant, whereas the residual direct path was not statistically significant. The significant negative path from perceived red tape to PSM supported H2, and the significant positive path from PSM to occupational commitment supported H3. The significant bootstrapped indirect effect further supported H4, indicating that PSM served as a statistically significant indirect pathway linking perceived red tape to occupational commitment.

In terms of effect size and practical relevance, the standardized path from PSM to occupational commitment was relatively strong (*β* = 0.541), indicating that PSM was the most substantial predictor in the model. By contrast, the standardized path from perceived red tape to PSM was smaller in magnitude (*β* = −0.177), suggesting that the motivational effect of red tape was statistically significant but modest. The model explained 11.2% of the variance in PSM and 35.2% of the variance in occupational commitment, indicating that the proposed model had meaningful explanatory power for occupational commitment, while other factors beyond perceived red tape and PSM may also contribute to PCPs’ professional attachment (see Table 3).

**Table 3:** Latent-variable SEM results for the mediation model.

Path / effect	*b*	SE	*p*	*β*	95% BC bootstrap CI
Red tape → PSM	−0.131	0.052	0.013	−0.177	[−0.241, −0.033]
PSM → Occupational commitment	0.313	0.068	<0.001	0.541	[0.189, 0.456]
Red tape → Occupational commitment	−0.048	0.031	0.125	−0.113	[−0.126, 0.001]
Indirect effect	−0.041	0.021	0.047	—	[−0.095, −0.010]
Direct effect	−0.048	0.031	0.125	—	[−0.126, 0.001]
Total effect	−0.089	0.041	0.031	—	[−0.192, −0.024]

Results were estimated using latent-variable SEM. b = unstandardized coefficient; β = standardized coefficient; BC bootstrap CI = bias-corrected bootstrap confidence interval based on 5,000 bootstrap samples. The model controlled for gender, age, work experience, professional title, staffing status, administrative position, education level, and marital status.

#### 3.1.6 Additional robustness checks

Several additional analyses were conducted to assess the stability of the quantitative findings. First, the observed-score mediation model produced results that were broadly consistent with the latent-variable SEM in terms of the directions of the main paths and the significance of the indirect effect. Perceived red tape was negatively associated with PSM (*b* = −0.103, SE = 0.036, *p* = 0.005; *β* = −0.173), PSM was positively associated with occupational commitment (*b* = 0.453, SE = 0.059, *p* < 0.001; *β* = 0.413), and the indirect effect was statistically significant (*b* = −0.047, SE = 0.019, *p* = 0.012, 95% BC bootstrap CI [−0.090, −0.015]). The observed-score mediation model produced results broadly consistent with the latent-variable SEM for the main paths and indirect effect; therefore, the indirect pathway was treated as more stable than the exact extent of mediation. Second, the nonlinear analysis showed that the negative linear association between perceived red tape and PSM remained significant. The quadratic term predicting PSM was positive but not fully robust when evaluated using the bootstrapped confidence interval, and the quadratic term for occupational commitment was not significant. Third, interaction-based heterogeneity analyses showed that the associations of perceived red tape with PSM and occupational commitment did not significantly differ by administrative position, staffing status, gender, or work experience. Fourth, the reverse-path sensitivity analysis did not support an alternative reverse mediation model from occupational commitment to perceived red tape through PSM, because the reverse indirect effect was not statistically significant (*b* = −0.120, SE = 0.114, *p* = 0.292, 95% BC bootstrap CI [−0.355, 0.117]). Overall, these analyses provided additional support for the main indirect pathway, although some results were model-dependent and the cross-sectional design precludes causal inference (see Table 4).

**Table 4:** Additional robustness checks.

Analysis	Key test	Estimate	SE	*p*	95% BC bootstrap CI	Conclusion
Observed-score mediation	Red tape → PSM	−0.103	0.036	0.005	[−0.177, −0.033]	Consistent with main SEM
Observed-score mediation	PSM → Occupational commitment	0.453	0.059	<0.001	[0.335, 0.570]	Consistent with main SEM
Observed-score mediation	Indirect effect	−0.047	0.019	0.012	[−0.090, −0.015]	Significant
Nonlinear analysis	Red tape^2^ → PSM	0.064	0.031	0.038	[−0.002, 0.120]	Limited evidence of nonlinearity
Nonlinear analysis	Red tape^2^ → Occupational commitment	−0.037	0.029	0.202	[−0.098, 0.017]	Not significant
Interaction-based heterogeneity	Interactions predicting PSM	—	—	0.284–0.984	All CIs included zero	No significant heterogeneity
Interaction-based heterogeneity	Interactions predicting occupational commitment	—	—	0.494–0.918	All CIs included zero	No significant heterogeneity
Reverse-path sensitivity analysis	Reverse indirect effect	−0.12	0.114	0.292	[−0.355, 0.117]	Reverse mediation not supported

*N* = 306. The observed-score mediation, nonlinear analysis, and interaction-based heterogeneity analysis used scale means. Perceived red tape was mean-centered before calculating squared and interaction terms. Interaction-based heterogeneity analyses tested administrative position, staffing status, gender, and work experience. CIs are bias-corrected bootstrap confidence intervals.

### 3.2 Qualitative findings

#### 3.2.1 Main forms of perceived red tape

Thematic analysis identified three main forms of perceived red tape among PCPs: regulatory constraints that did not fit clinical work, inefficient and repetitive documentation and record-keeping requirements, and administrative arrangements detached from clinical work. Administrative tasks were interpreted as perceived red tape when participants described them as burdensome, repetitive, weakly connected to clinical goals, or misaligned with patient care. The first form referred to rules and regulations that were poorly adapted to primary healthcare and that constrained clinical judgment and professional autonomy. The second involved repetitive recording, reporting, and documentation, whereas the third covered low-value meetings, inspections, and related administrative arrangements that disrupted clinical work and consumed time and energy. To better present the findings, Table 5 summarized the three forms, their descriptions, specific manifestations, and representative interview excerpts.

**Table 5:** Main forms of red tape perceived by PCPs.

Main form	Description	Specific examples	Representative interview excerpts
Regulatory constraints that do not fit clinical work	Some rules and procedures embedded in clinical practice were poorly adapted to primary care. They were perceived by PCPs as high compliance burdens and partly limited professional judgment and clinical autonomy.	Health insurance restrictions (total cost control, medication scope, drug types, and indication limits); compliance review pressure	Interviewee LCDA: “Health insurance imposes strict limits on medication. For example, omeprazole can only be used if patients have swallowing difficulties. But some patients cannot take oral medication due to vomiting, even without swallowing problems. Clinically, the drug is needed. However, according to insurance rules, prescribing outside indications may be punished. So many times we do not dare to prescribe it.” Interviewee YTHY: “Sometimes new symptoms appear after a patient is hospitalized. Clinically, we may need to conduct tests. If no problem is found, it may be considered overtreatment. If we do not test and later a problem arises, we are asked why we did not test. This puts us in a difficult position.”
Inefficient and repetitive documentation and record-keeping	Repetitive recording, reporting, and documentation requirements from different departments or systems were perceived as increasing workload but had limited practical value.	Form filling and reporting; repeated entries across multiple systems; excessive record-keeping	Interviewee YTLY: “We need to upload data to the medical supervision system, the public health system, and the cloud platform management system. All three require the same data. It is very tedious.” Interviewee YTFS: “We have to handle family doctor contract work and fill many forms each year. Residents may not even use these forms. Although the system has electronic records, we still need paper copies. It wastes human resources and energy. It feels unnecessary.”
Administrative arrangements detached from clinical work	Certain meetings, inspections, and other administrative tasks were perceived as weakly related to clinical work but consumed a large amount of clinical time and energy.	Low-value meetings; supervision and inspection tasks; preparation for inspections	Interviewee LCSX: “Sometimes higher-level authorities come to inspect. We need to prepare materials, attend meetings, or cooperate with investigations. These tasks take time. Inspections have limited impact on work and make us feel busy coping. We have to leave our posts to handle these tasks, which reduces patient consultation time.”

Themes were derived from thematic analysis of semi-structured interviews. Excerpts are representative examples.

#### 3.2.2 Qualitative analysis of the mediating role of PSM

The qualitative data further clarified how perceived red tape was linked to occupational commitment through PSM. In the interviews, PCPs’ PSM was mainly reflected in their willingness to relieve patients’ suffering, provide useful clinical services, and realize the value of serving patients and the public. However, participants’ accounts suggested that perceived red tape made it more difficult for some PCPs to maintain this public-serving orientation. This process was reflected in three connected experiences: constrained clinical autonomy, reduced professional competence, and weakened relatedness to patients and public-serving work.

First, perceived red tape constrained PCPs’ need for autonomy by limiting their ability to make clinical decisions according to patients’ actual conditions. Some participants described health insurance rules, medication restrictions, and compliance requirements as forcing them to consider administrative consequences before making treatment decisions. In these situations, clinical judgment was no longer experienced as a fully professional and patient-oriented decision, but as a decision constrained by external rules and possible penalties.

*Interviewee YTHY*: “We just want to focus on seeing patients and devote our energy to patient care. But in reality, I not only have to handle clinical work, but also take on many administrative tasks that feel of little use to us. Even during consultations, I have to keep track of health insurance limits in my mind. It is hard to truly focus on patient care. Over time, I feel less motivated in my work.”

Second, perceived red tape obstructed physicians’ need for competence by occupying clinical time and consuming work-related energy. Some PCPs reported that they were required to handle a substantial volume of non-clinical tasks, such as repetitive reporting, preparation for inspections, and extensive form-filling, thereby reducing the time and resources available for clinical diagnosis and treatment. Under such conditions, their perceived sense of competence in professional practice is weakened, leading to a subjective feeling of being unable to perform their core clinical duties effectively, as well as a diminished perception of their capacity to contribute meaningfully to the public interest.

*Interviewee ZBBS*: “Reporting and preparing for inspections take up a large part of my work. These tasks are very tedious. Only a limited amount of time is left for clinical work. I feel that I am not really doing my proper job. I used to want to be someone who contributes to society, but now my motivation is just to hold on until retirement.”

Third, perceived red tape disrupted the relational meaning of clinical work. Some PCPs reported that administrative demands, meetings, and inspections interrupted clinical interactions and reduced the time available for patients. In addition, health insurance regulations often transformed consultations into repeated explanations of compliance requirements. Physicians had to manage patients’ expectations while also avoiding administrative non-compliance. As a result, patient care could shift away from a trust-based helping relationship toward a more defensive and compliance-oriented interaction.

*Interviewee LCDC*: “To avoid offending the patient and also avoid violating the health insurance prescription rules, you have to explain patiently and carefully. Sometimes you have to explain in a very humble way.”

The frustration of the three basic psychological needs may be associated with motivational externalization among some PCPs. When PCPs repeatedly encountered rules and administrative tasks that were perceived as high in burden but weakly connected to clinical value, they found it harder to experience their work as a self-endorsed way of serving patients and the public. For some participants, this was associated with a shift from public-serving motivation toward a more passive or instrumental work orientation.

*Interviewee LCDC*: “At the beginning, I intended to serve the people. However, there are so many meetings and reports, along with a lot of disorganized and miscellaneous tasks, and the remuneration is not high. At this point, it is no longer enough to just talk about slogans; the starting point of the work still needs to be more realistic.”

The interviews also helped explain how lower PSM was associated with weaker occupational commitment. When PCPs maintained stronger public-serving motivation, they tended to describe medical work as a profession through which they could realize personal value and contribute to patients, even when external incentives were limited. This sense of meaning supported emotional attachment to the profession and willingness to remain engaged.

*Interviewee LCSX*: “I believe that being a doctor means working in a down-to-earth way to relieve patients’ suffering through actual practice. Although I could leave this institution and take a higher-paying job, such as becoming a massage therapist, I do not think it is necessary. I identify more with this way of steadily serving patients. If my child is willing, I would support them in becoming a doctor.”

In contrast, when PSM was weaker, participants tended to evaluate the medical profession more through external conditions such as income, workload, career prospects, and family responsibilities. Because primary healthcare positions often offered limited financial rewards and development opportunities, this externally oriented evaluation was associated with weaker occupational attachment and a lower willingness to recommend the profession to the next generation.

*Interviewee FSDT*: “What keeps me in this profession is mainly to support my family. If my child performs well in school, I would not want them to study medicine. I would prefer them to choose a field and career with better prospects. In my view, being a doctor does not provide high income and offers limited opportunities for development. So if there is a better option, there is no need to follow this path.”

Taken together, the qualitative findings suggest that perceived red tape may weaken PSM by constraining PCPs’ ability to experience clinical work as autonomous, competent, and relationally meaningful public service. Lower PSM, in turn, may make occupational commitment more dependent on external conditions such as income, workload, and career prospects.

### 3.3 Integration of quantitative and qualitative findings

Table 6 integrated the quantitative results of the SEM with the qualitative findings. The quantitative results indicated that perceived red tape was negatively associated with PSM, that PSM was positively associated with occupational commitment, and that the indirect effect of perceived red tape on occupational commitment through PSM was statistically significant. The qualitative interview data helped elaborate this pathway by showing that perceived red tape was experienced as constraining autonomy, competence, and relatedness among PCPs, thereby contributing to the externalization of motivation and a weakening of PSM. When PSM was undermined, PCPs tended to evaluate the medical profession more in terms of external incentives. However, given the relatively unfavorable external conditions in primary healthcare settings, this externally oriented evaluation was associated with a reduced level of occupational attachment, which may in turn manifest as lower occupational commitment.

**Table 6:** Joint display of quantitative and qualitative findings.

Quantitative finding	Qualitative finding	Type of integration	Integration inference
Perceived red tape was negatively associated with PSM (*b* = −0.131, *p* = 0.013; *β* = −0.177).	Perceived red tape constrained the clinical autonomy of PCPs, weakened their sense of professional competence, and disrupted the relational meaning of clinical work, making it difficult for them to maintain a public-interest-oriented professional orientation.	Elaboration	Perceived red tape was associated with frustration of the three basic psychological needs in self-determination theory (autonomy, competence, and relatedness), thereby weakening PSM.
PSM was positively associated with occupational commitment (*b* = 0.313, *p* < 0.001; *β* = 0.541).	PCPs with stronger PSM tended to view medical work as a public-value-oriented profession and thus show higher occupational commitment. In contrast, those with weaker PSM rely more on external factors to evaluate their work; however, limited external incentives in primary healthcare lead to lower professional evaluations and, consequently, weaker occupational commitment.	Convergence and elaboration	When PSM is undermined, individuals tend to shift their evaluation of the profession toward external incentives; when such external conditions fail to meet their needs, this may result in lower occupational commitment.
The indirect effect of perceived red tape on occupational commitment through PSM was statistically significant (*b* = −0.041, *p* = 0.047, 95% BC bootstrap CI [−0.095, −0.010]).	Some PCPs, despite experiencing high levels of perceived red tape, are able to maintain their PSM and occupational commitment due to protective factors such as family socialization and policy endorsement. In contrast, another group of PCPs exhibits lower occupational commitment, which is not attributable to a decline in PSM but rather to risk factors such as low job satisfaction and frequent physician–patient conflicts.	Elaboration and divergence	The significant indirect pathway represents an average-level mechanism. Qualitative divergence suggests that this pathway may be conditioned by protective and risk factors not included in the quantitative model.

This joint display integrates quantitative SEM results and qualitative themes using triangulation. BC bootstrap CI = bias-corrected bootstrap confidence interval.

## 4 Discussion

This study adopted a convergent parallel mixed-methods design to examine the relationships among PCPs’ perceived red tape, PSM, and occupational commitment. The quantitative findings showed that perceived red tape was negatively associated with PSM, that PSM was positively associated with occupational commitment, and that PSM statistically mediated the association between perceived red tape and occupational commitment. The qualitative findings indicated that PCPs mainly faced three forms of red tape and further clarified how PSM functioned in this relationship. These findings suggest that red tape is not only an institutional burden, but may also be associated with lower occupational commitment by weakening PCPs’ experience of, and identification with, the public service value of their work.

This study found that PCPs perceived a moderate level of red tape (M = 8.21, full score = 15), suggesting that this issue warrants attention. The qualitative data showed that this perception mainly took three forms: regulatory constraints that did not fit clinical work, inefficient and repetitive documentation and record-keeping requirements, and administrative arrangements detached from clinical work. Institutional theory argues that institutions are not merely external constraints; they also define, through rules, norms, and shared meanings, how work should be performed and what kinds of work are regarded as legitimate within organizations (13). In healthcare settings, professional norms and occupational meaning usually assign PCPs professional roles related to public service values, such as clinical judgment and patient care. However, to demonstrate the legitimacy of organizational behavior, primary healthcare organizations often need to implement regulatory requirements, documentation tasks, and administrative meetings designed to control costs, monitor quality, and prevent risks. Kaufman’s observation that “one person’s red tape may be another person’s safeguard” helps explain this institutional tension (29). PCPs rarely have opportunities to participate in policymaking and can only passively implement measures that regulators regard as necessary safeguards (30). Under such circumstances, tension arises between the compliance-proving logic of administrative rules and the professional service logic of primary healthcare work. As a result, PCPs may experience these requirements as institutional burdens that restrict professional autonomy, consume clinical time, and have only weak relevance to improving patient well-being, thereby constituting perceived red tape.

From an international comparative perspective, red tape arising from conflicts between administrative compliance logic and professional service logic takes different forms across governance structures, mainly because of differences in rule-generating logics and accountability structures (11). The forms of red tape revealed in this study represent manifestations of primary healthcare red tape under a state-level, control-oriented governance structure. In this type of governance, red tape mainly derives from hierarchical accountability requirements, such as superior-level regulation, health insurance audits, inspections, and performance assessments. In more pluralistic regulatory systems, such as the United States, red tape may be more likely to appear in the form of prior authorization, payer-specific documentation, payment rules, and compliance requirements (31). In performance-accountability systems, such as the United Kingdom, red tape may be reflected in quality indicator assessment, performance reporting, audit evaluation, and requirements for proof-based materials (32). The cross-national differences mainly lie in the institutional sources of red tape; the common mechanism is that, when certain rules and procedures are experienced by frontline physicians as high in burden and low in clinical value, necessary regulation may be transformed into an institutional burden.

One core finding of this study is that PSM statistically mediated the association between PCPs’ perceived red tape and occupational commitment. The qualitative data showed that some PCPs spent a large amount of time on red tape tasks that were weakly related to clinical work, low in value, and heavy in burden. This not only constrained their professional autonomy, but also weakened their perceived professional competence and may have undermined the relational meaning embedded in physician–patient interactions. From the perspective of self-determination theory, these burdens appeared to obstruct PCPs’ three basic psychological needs for autonomy, competence, and relatedness. When these basic psychological needs are frustrated, motivational externalization may occur (33), which is manifested among PCPs as a lower level of PSM. A decline in PSM may make PCPs more likely to evaluate the medical profession through external conditions such as income. However, because primary healthcare institutions often provide relatively limited external incentives, this may weaken individuals’ emotional attachment to the profession and their willingness to remain engaged, which may be associated with a lower level of occupational commitment. Conversely, PCPs with stronger PSM may continue to pursue public interest goals and maintain a higher level of occupational commitment even under considerable institutional pressure. Thus, PSM is a key psychological mechanism linking red tape and occupational commitment and should be protected and strengthened in the process of primary healthcare reform.

The results showed that PCPs’ occupational commitment was at a moderate-to-high level (M = 30.37, full score = 40), suggesting that this group has a certain degree of occupational resilience. However, this result should be interpreted with caution. Occupational commitment is not a single form of positive professional identification. It may reflect individuals’ emotional attachment to, and value identification with, the profession, but it may also include continuance commitment arising from high leaving costs, limited alternative opportunities, and difficulty in giving up existing investments (6, 34). In this study, most PCPs had a bachelor’s degree or below, younger respondents accounted for a relatively small proportion, and opportunities for occupational mobility were limited. Career change was often accompanied by high sunk costs and switching costs. In this context, even if individuals’ evaluations of the profession decline, they may not necessarily leave the medical profession. Therefore, the current moderate-to-high level of occupational commitment may not fully mean that PCPs “actively want to stay”; it may also partly reflect the reality that they “have to stay.” Such passive retention is more deeply rooted in switching costs and restricted external opportunities than in stable identification with, and attachment to, the medical profession. In the long term, passive retention may lead to increased occupational burnout and reduced professional engagement among PCPs, thereby impairing the efficiency and quality of primary healthcare services (35). The level of occupational commitment revealed in this study should be viewed as a proximal psychological indicator of PCPs’ retention status rather than sufficient evidence of long-term stability. Future research needs to adopt longitudinal designs to further examine the dynamic relationships among red tape, PSM, occupational commitment, and long-term outcomes.

Although the quantitative and qualitative findings showed considerable consistency in overall patterns, the qualitative data revealed more complex phenomena that were not fully captured by the quantitative model. The qualitative analysis found that even PCPs who described heavy perceived red tape did not always speak of weaker motivation or less attachment to their profession. Interview data indicated that these individuals were often supported by protective factors such as family socialization, policy identification, local social ties, and organizational support. These factors appeared to buffer the negative effects of red tape to some extent. In addition, some PCPs showed lower occupational commitment, but this did not appear to result directly from red tape or from diminished PSM. Instead, it seemed more closely related to risk factors such as low job satisfaction, frequent doctor–patient conflicts, and limited career development opportunities. Future research in primary healthcare settings may incorporate these protective and risk factors into the analytical framework and further examine whether they constitute boundary conditions for the effects of red tape on PSM and occupational commitment.

The theoretical contributions of this study are mainly reflected in two aspects. First, this study introduced red tape as a form of institutional pressure into research on occupational commitment among PCPs, thereby extending previous research perspectives that mainly explained occupational commitment from the standpoint of individual characteristics and organizational support. The findings suggest that PCPs’ occupational commitment is influenced not only by personal motivation and organizational environment, but also by the degree of fit between administrative rules, compliance requirements, and the professional service logic. Second, by integrating institutional theory and self-determination theory, this study explained why PCPs perceive red tape and how red tape may be associated with PSM and occupational commitment by obstructing the satisfaction of the three basic psychological needs. In this way, the findings deepen psychological-level understanding of how institutional environments shape PCPs’ work-related attitudes.

From a practical perspective, the findings of this study suggest that reducing the effects of red tape does not mean weakening necessary regulation. Rather, policy reforms should prioritize optimizing institutional arrangements that PCPs perceive as highly burdensome, low in value, and disconnected from core clinical goals. Specifically, first, primary-care adaptability assessments should be introduced into the design of policies related to health insurance management, medication restrictions, clinical testing standards, and public health assessment, so as to avoid rigid “one-size-fits-all” rules that excessively compress physicians’ space for professional judgment. Second, institutionalized channels should be established for PCPs to participate in policy design, pilot feedback, and rule revision, enabling frontline implementers to provide timely feedback on which requirements have practical clinical value and which mainly increase useless compliance burdens. Third, information system integration, process reengineering, and data sharing should be promoted to reduce repeated entry across multiple platforms, the parallel use of paper materials and electronic systems, repeated record-keeping, and proof-based assessments. Fourth, meetings, supervision, and inspections should be optimized, and the focus of assessment should shift from completeness of materials and process record-keeping to clinical quality, continuity of care, patient experience, and real health outcomes. Fifth, primary healthcare institutions should also improve their internal division of labor by reducing the proportion of non-medical tasks undertaken by physicians through administrative assistance, team collaboration, and process optimization. Only when PCPs have more time, capacity, and autonomous space to devote to patient care can their PSM and occupational commitment be continuously maintained.

This study has several limitations. First, this study adopted a cross-sectional design. Although reverse-path sensitivity analysis and robustness checks were conducted, strict causal inference cannot be made. Future research could use longitudinal follow-up, quasi-experimental designs, or experience sampling methods to further examine the dynamic relationships among red tape, PSM, occupational commitment, and long-term outcomes. Second, this study mainly relied on self-reported data, which may be affected by social desirability bias, common method bias, and perception bias. Future research could combine subjective and objective multi-source data to assess red tape and its consequences more objectively. Third, the sample was mainly drawn from primary healthcare institutions in Shandong Province, and the external validity of the findings still needs to be further examined across different regions, different types of primary healthcare institutions, and different governance contexts. Future studies could adopt cross-regional, cross-institutional, or cross-national comparative designs to examine the forms and mechanisms of red tape under different governance structures. Fourth, in the qualitative interviews, PSM was mainly inferred indirectly from respondents’ accounts of work meaning and service motivation. Future research could design more targeted interview guides to achieve more accurate assessment.

## 5 Conclusion

This study found that PCPs perceived a moderate level of red tape, the main forms of which included regulatory constraints that did not fit clinical work, inefficient and repetitive documentation and record-keeping requirements, and administrative arrangements detached from clinical work. The quantitative analysis showed a significant negative total effect between perceived red tape and occupational commitment, and PSM played a mediating role in this pathway. The qualitative analysis further suggested that perceived red tape was experienced as constraining autonomy, competence, and relatedness, which may help explain why it was linked to weaker PSM and lower occupational commitment.

The contributions of this study are mainly reflected in three aspects. Theoretically, this study introduced red tape as a form of institutional pressure into research on occupational commitment among PCPs, thereby extending previous research perspectives that mainly explained occupational commitment from individual and organizational levels. Methodologically, this study adopted a convergent parallel mixed-methods design, combining statistical testing through SEM with mechanism interpretation based on qualitative interviews, thereby enhancing understanding of the relationships among variables and the processes through which they occur in practice. Empirically, based on the Chinese primary healthcare context, this study identified the specific forms of red tape perceived by PCPs and revealed the role of PSM in the relationship between red tape and occupational commitment.

This study has several limitations related to its cross-sectional design, self-reported measures, and single-province sample. Future research should adopt longitudinal, multi-source, and multi-regional designs to further examine the effects of red tape on long-term outcomes such as occupational commitment and occupational burnout. In terms of policy, primary healthcare reform should not simply reduce regulation. Instead, it should prioritize the governance of institutional arrangements that are high in burden, low in value, and disconnected from core clinical goals. By improving the primary-care adaptability of rules, establishing mechanisms for PCPs to participate in policy feedback, integrating information systems, reducing repeated reporting, optimizing assessments and inspections, and protecting physicians’ clinical time, reform efforts can support the long-term maintenance of PSM and occupational commitment among PCPs.

## Data Availability

The de-identified quantitative data supporting the conclusions of this article will be made available by the corresponding author upon reasonable request. The full qualitative interview transcripts are not publicly available because they may contain contextual information that could compromise participant confidentiality.
